# Combination immunotherapy: Where do we go from here?

**DOI:** 10.1186/s40425-015-0083-z

**Published:** 2015-08-18

**Authors:** Abigail E. Overacre, Sema Kurtulus, Mario Sznol, Drew M. Pardoll, Ana Anderson, Dario A. A. Vignali

**Affiliations:** Department of Immunology, University of Pittsburgh, Pittsburgh, PA 15213 USA; Evergrande Center for Immunologic Diseases and Ann Romney Center for Neurologic Diseases, Brigham and Women’s Hospital and Harvard Medical School, Boston, MA 02115 USA; Division of Medical Oncology, Yale Comprehensive Cancer Center, Yale University, New Haven, CT 06250 USA; Division of Oncology, Johns Hopkins University School of Medicine and Sidney Kimmel Comprehensive Cancer Center, Baltimore, MD 21287 USA

**Keywords:** Immunotherapy, Cancer, T cells, Therapy, Exhaustion, Immunology

## Abstract

The remarkable clinical success of cancer immunotherapies targeting the checkpoint receptors CTLA-4 and PD-1 has generated considerable excitement and emboldened efforts to build on this important foundation. Research efforts are now focused on understanding the mechanism of action of these immunotherapies, identifying new inhibitory mechanisms that could be targeted to achieve responses in patients with refractory cancers, and developing approaches that might exhibit efficacy against “immunologically inert” tumors. The outstanding challenges in moving forward are developing reliable strategies for determining which patients will respond optimally to a given immunotherapy, and what combination of immunotherapies and conventional therapies will prove beneficial against each tumor type. These issues were discussed in a one-day workshop at the SITC meeting in November 2014.

## Beyond the inflamed/non-inflamed classification of the immune microenvironment

This session highlighted new immune-based approaches to classify tumors with the aim of improving prognostic power. Jerome Galon (INSERM, France) presented the Immunoscore, an immune-profiling approach that uses four parameters to determine an immune contexture for a given tumor sample. The four parameters account for T cell type (CD8) and function (the CD45RO memory marker), cell density, and location (invasive margin or center) of the immune cells within tumor tissue. Immunoscore data collected thus far indicate that a high immunoscore correlates strongly with better prognosis. Immunoscore profiling has been validated initially in colorectal carcinoma and currently a worldwide task force is investigating its application to many other cancer types. Toni Ribas (UCLA, USA) presented data showing the application of components of immunoscore profiling to melanoma. Scoring for the presence of CD8, CD4, PD-1 and PD-L1 among responders and non-responders to checkpoint blockade therapy showed that the best predictors of response were the presence of CD8^+^ T cells in the invasive margin and expression of PD-1 and PD-L1. Suzanne Topalian (Johns Hopkins, USA) expanded on the theme of PD-1/PD-L1 expression as a predictor of response. She pointed out that despite the use of various antibodies in different studies, all have shown that a patient is 2–3 times more likely to respond if pre-treatment biopsies exhibit PD-L1 expression. Interestingly, PD-L1 expression is often observed at the tumor margin as if providing a “shield” to disarm infiltrating T lymphocytes. However, she also emphasized that not all patients that responded well to PD1/PD-L1 targeted therapy had PD-L1+ biopsies. She presented data showing that PD-L1 expression may vary among melanoma lesions from the individual patient or may be present at focal sites of tumor lesions, raising the possibility that some PD-L1+ tumors/patients may be missed due to sampling error. Therefore, using PD-L1 expression as a “biomarker” to select patients for anti-PD-1 therapy may result in exclusion of patients that would benefit from therapy. In some patients receiving combined anti-CTLA-4 and PD-1, it has been noted that therapy can drive T cells to the tumor site and increase T cell production of IFN-γ which in turn can induce PD-L1 expression by tumor cells. Thus, T cell infiltration and PD-L1 expression within tumor tissue can be dynamic and other markers are needed to select patients for therapies.

## What does an “immunologically inert” tumor mean?

This session highlighted approaches to treating tumors that lack a T cell infiltrate. This could be due to a lack of recognizable tumor antigens. Previously, the most successful immunotherapies have utilized checkpoint blockade; however, immunologically inert tumors are resistant to this therapy. Bob Schreiber (Washington University, USA) emphasized the importance of identifying mutant antigens in edited versus non-edited tumors. He presented data on the use of exome sequencing to identify tumor antigens that have or have not undergone immunoediting. By comparing mutant proteins of unedited regressor tumors to those from edited progressor tumors, personalized cancer vaccines have been developed that trigger T cell responses against tumor-specific mutant antigens and showed that these therapies are as effective as checkpoint receptor blockade. These mutant antigens can indeed alter the immunogenicity of the tumor, such as the R913L mutant Spectrin-β2, which drastically increases the immunogenicity of d42m1 sarcoma. Tom Gajewski (University of Chicago, USA) presented research on determining better targets for non-infiltrated tumors through innate immune sensing, allowing for better priming of the adaptive immune response. His laboratory identified that STING, an intracellular membrane tethered protein, could be a potential therapeutic target for immunologically inert tumors. The STING pathway triggers immune responses via a tumor cell:DC interaction and pre-clinical studies showed that STING agonists greatly reduce tumor growth. Gajewski also mentioned the importance of β-catenin signaling. When this pathway is active, T cells are excluded, altering the microenvironment. Previous work has shown that cell death in the tumor microenvironment can also be a critical component of activating the innate immune response through the release of endogenous adjuvants or defensins. Michael Lotze (University of Pittsburgh, USA) stressed that an important factor to take into account in treating tumors that have developed resistance to cytotoxic chemotherapies is cancer cell death. The tumor microenvironment is highly hypoxic and this can lead to altered expression of autophagy molecules, such as HMGB1. Therefore, modulation of HMGB1-driven autophagy and DAMP release may be a strategy for improving responses to chemo-resistant tumors. Jennifer Wargo (MD Anderson Cancer Center, USA) presented data showing that by combining novel targeted therapies with more classical ones, such as BRAF inhibitors, melanomas can actually become more immunogenic through enhanced expression of tumor antigens and tumor cell death. With this theme in mind, Jennifer Wargo and colleagues combined targeted therapy with immunotherapy and found that the combination of BRAF inhibitors and anti-PD-1 led to a reduction in melanoma growth, as well as an increase in CD8^+^ effector T cells in the tumor microenvironment. Positive responses were also shown in other murine tumor models, such as Gastrointestinal Stromal Tumor (GIST). Clinical trials are currently underway utilizing this approach.

## How to break tumor-induced immune suppression

This session highlighted mechanisms of immune suppression in tumors and strategies for targeting them. Dario Vignali (University of Pittsburgh, USA) discussed the role of regulatory T cells (T_regs_). He showed a striking reduction in tumor growth by decreasing T_reg_ stability and function through genetic deletion or blockade of the surface receptor, Neuropilin-1 (Nrp1), while maintaining immune homeostasis. Indeed, mice lacking Nrp1 on their T_regs_ survive long term without inducing systemic autoimmune inflammatory reactions. He showed that targeting the T_reg_-derived inhibitory cytokine interleukin-35 (IL35) also limited tumor growth. Ana Anderson (Harvard Medical School, USA) presented data on TIGIT, a novel checkpoint receptor that binds to nectin and nectin-like ligands, and its role in regulating anti-tumor responses. She showed that TIGIT restrains anti-tumor CD8^+^ T cell responses and could be a promising target for combinatorial blockade together with Tim-3. She emphasized that as the number of checkpoint receptors grows, it is critical to achieve a greater understanding of the unique function of each checkpoint receptor in order to inform the rational application of combinatorial immunotherapy approaches. Susan Kaech (Yale School of Medicine, USA) presented data that BRAF inhibitors can trigger immune response by increasing IFN-γ production and CD40L expression by T cells and consequently activating macrophages within the tumor. While this is advantageous, BRAF inhibitors also caused an increase in PD-1 expression, emphasizing the requirement for combinatorial approaches. John Wherry also focused on the importance of combinatorial approaches. He pointed out that CD8^+^ T cells within the tumor can be divided into two major subpopulations: Eomes^Hi^PD-1^Hi^, and Tbet^Hi^PD-1^Lo^. He underscored that the changes in the Eomes^Hi^PD-1^Hi^ subset could be useful in predicting the response to immunotherapy in combination with radiation. He emphasized that while these cells are considered “exhausted”, they retain some function and could contribute to control of tumor growth. He also pointed out that PD-1 knockout cells can still express high levels of other checkpoint inhibitors which underlines the potential benefit of combination immunotherapy.

## Ideas for innovative combination approaches in the future

This session focused on novel combinatorial approaches in the clinic. Hassane Zarour (University of Pittsburgh, USA) highlighted Tim-3 and BTLA as potential partners for anti-PD-1 therapy. He also stressed the potential utility of anti-IL-10 therapy as he showed that CD8^+^ T cells increased IL-10 receptor expression after PD-1 blockade. Steve Hodi (Dana-Farber Cancer Institute, USA) presented data combining anti-CTLA-4 with cytokines, such as GM-CSF, or anti-angiogenesis treatments, such as VEGF blockade. He underscored the importance of understanding the mechanisms induced by these combined therapies may allow for improvement in efficacy. Mario Sznol (Yale Cancer Center, USA) gave a provocative talk in which he referred to immunotherapy as “imprecision medicine”. He cited all the ongoing permutations of immunotherapy approaches in the clinic and how little we actually know about how any of these work.

## Conclusions

While promising immunotherapies are now in the clinic with an encouraging pipeline of new targets under development, considerable work lies ahead in order to determine the most effective therapeutic combinations for each tumor type and individual patients. In this workshop, the potential advantages and challenges of combination immunotherapy in pre-clinical models and clinical trails was evaluated. The enhanced therapeutic effects of combining anti-CTLA-4 and anti-PD-1 in clinical studies have reinforced the value and importance of continued development of new immunotherapies against other inhibitory molecules and testing additional combinatorial approaches. It is also critical to understand the mechanisms induced by each immunotherapy or combination of therapies in order to identify potential biomarkers and determine how to optimize therapies. Lastly, in order to increase the response rate to immunologically inert tumors, it is important to understand and take advantage of the mechanisms that drive immune cell infiltration into the tumor. Such data will be critical to the rational development of combinatorial immunotherapies in the future.

## Abbreviations

PD1: Programmed cell death 1
PDL1: Programmed cell death ligand 1
CTLA4: Cytotoxic T-lymphocyte-associated protein 4
STING: Stimulator of interferon genes
HMGB1: High mobility box 1
Nrp1: Neuropilin-1

